# Updated Static Influential Factor Analysis for Unidirectional Carbon-Based Composites

**DOI:** 10.3390/polym16182642

**Published:** 2024-09-19

**Authors:** Bae Jun Kwon, Chan-Jung Kim

**Affiliations:** 1 Stability, DNV AS Norway, 1363 Oslo, Norway; bae.jun.kwon@dnv.com; 2 School of Mechanical Engineering, Pukyoung National University, Busan 48513, Republic of Korea

**Keywords:** carbon fiber orientation, modal participation factor, modal parameters, revised static load influential factor, uniaxial static load, unidirectional carbon-based composite structure

## Abstract

The orientation of carbon fibers significantly affects the dynamic properties of unidirectional carbon-based composites (UCBCs), with variations under different static loads. A previous study analyzed changes in the modal parameters of UCBC structures by using the static load influential factor (SLIF). This study introduces a revised SLIF, derived from a simplified formulation that accounts for shifts in resonance frequency and the in-phase relationship between static load and modal response. The revised SLIF is theoretically linked to the modal participation factor in UCBC structures. The dynamic behavior of UCBCs was studied across six modes—four bending and two torsional—using specimens with five carbon fiber orientations, from 0 to 90 degrees. The revised SLIF showed significant effects in two robust specimens, #1 and #2, and an isotropic SUS304 specimen subjected to uniaxial pre-static load, with resonance frequency variations under 0.16%. In contrast, the original SLIF gave negligible results in the fifth mode due to a damping term, which, when multiplied by the resonance frequency, led to an undetectable indicator. Therefore, the revised SLIF more effectively captures the static load’s impact on UCBC dynamic behavior compared with the original method.

## 1. Introduction

The merit of carbon-based composite structures lies in their high specific structural rigidity relative to their volume and mass and their decreasing manufacturing costs due to increased mass production in the mechanical industry. The mechanical properties of these composites display anisotropic behavior due to the arrangement of carbon fibers, prompting the proposal of various weaving methods, such as plain, twill, and unidirectional weaves [1,2,3]. Consequently, numerous mechanical characteristics, including thermodynamics, statics, and dynamics, have been examined, with this study focusing primarily on the dynamic behavior of the composite structure. The frequency response function (FRF) is extensively employed in the mechanical field to understand the dynamics of a target system, provided the system can be assumed to be linear [4,5,6]. By applying modal testing techniques to the FRFs, the dynamics of the system can be distinctly identified within the frequency domain through modal parameters, which include the resonance frequency, modal damping ratio, and corresponding mode shape vector [4,5,6]. The unidirectional layout of carbon fibers is fundamental to understanding the dynamics of carbon-based composites; therefore, it has been frequently utilized in studies involving unidirectional carbon-based composite structures (UCBCs). The dynamics of UCBCs have been explored through modal testing and subsequent analysis using estimated modal parameter information [7,8,9,10,11]. It has been previously established that the modal model of UCBCs is valid provided that the approximated model remains within acceptable error limits. The validation process for modal parameters can be conducted by using several criteria, including the coherence function of FRFs and the modal assurance criterion of mode shape vectors [4,5,6].

Earlier studies have demonstrated the reliability of the modal parameters of UCBC structures, which can be derived according to the modal parameter validation process. These criteria provide essential guidance for monitoring variations across multiple modes of UCBC specimens with different carbon fiber orientations [7,8,9,10,11]. Furthermore, the linearization of the UCBC model, characterized by modal parameters and the integration of two sub-components—the unidirectional carbon fibers and the binding matrix—can be represented in either series or parallel combinations within the overall system parameters, including stiffness and damping matrices [12,13,14].

The mechanical interplay with adjacent components is crucial to the dynamics of UCBC structures, with static loads applicable only to neighboring parts under fixed constraints. Several studies have focused on the behavior of carbon-fiber composites under static load conditions. Both static and quasi-static loading scenarios were applied to carbon-fiber composites, and their behavior under these conditions was analyzed [15,16,17,18]. Additionally, a damage identification technique was proposed for these composites under static loading [19]. Variations in electrical conductivity [20] and energy absorption [21] in carbon-fiber composites have also been evaluated under static loading conditions. Additionally, the effect of pre-static load was considered in joint mechanisms [22] and integrated optical fiber sensors [23] in carbon fiber composites. The orientation of carbon fibers is a key parameter influencing the UCBC structure, recently investigated through the static load influential factor (SLIF) [24]. The SLIF quantifies the impact of static load on the UCBC structure, defined as the ratio of the decoupled modal response to the mass-normalized static load. This influential factor involves the indirect inspection of variations in the dynamics of the target system, distinguishing it from direct nondestructive testing methods, such as those using eddy current sensors, acoustic emission sensors, or others [25,26,27]. The SLIF is defined as the specific modal column vector divided by the mass-normalized static load. Therefore, the value of the SLIF is proportional to the dynamic response at the resonance frequency of interest with respect to the assigned normalized static load, making it an efficient indicator for determining the sensitivity of the dynamic behavior of a target structure under pre-applied static load under a given boundary condition. According to the SLIF values for UCBC specimens with various carbon fiber orientations, higher SLIF values are typically observed in specimens that robustly withstand the assigned uniaxial static load, aligning closely with or with minimal deviations from the fiber orientation. However, the original SLIF, which incorporates all system matrices including both damping and stiffness, becomes less perceptible at high frequencies due to an increase in the modal damping term multiplied by the resonance frequency. Given that static loads are applied as real values at fixed locations, the decoupled modal response should also remain a real value under static loading conditions. Consequently, a revised SLIF is proposed to better assess the dynamic impact on the UCBC structure, considering only the real terms, which correspond to the stiffness matrix. This revised SLIF is a simplified formula yet effectively captures variations in the modal parameters of the UCBC structure under static loading. Notably, the revised SLIF is proportional to the modal participation factor of the structure. Results obtained by using the revised SLIF demonstrate consistent findings in low-frequency ranges but provide enhanced visibility and more significant results at higher frequencies by focusing solely on the stiffness system matrix. Therefore, the advancements of the revised SLIF include correcting the underestimated effect of static load in the high-frequency range and enhancing the understanding of the SLIF value as the modal participation factor at the resonance frequency of interest. The proposed method can be applied to isotropic structures as well as anisotropic ones, including UCBCs, because the modal parameters of the target system are the only necessary data for calculating the SLIF. However, the feasibility of linearizing the dynamic behavior is a mandatory condition for calculating the SLIF, as the modal parameters are obtained under the assumption of a linearized model.

The structure of this article can be summarized as follows: Section 2 presents the theory of the revised SLIF, which is formulated by using the linearized model of UCBC structures and is compared with the previous SLIF. Section 3 presents the modal parameters of UCBC specimens with five different carbon fiber orientations, measured by using experimental modal test techniques. Both the original and revised SLIFs were calculated, and their comparison is presented in Section 4, where the physical implications regarding the UCBC structure are also discussed. The main achievements are addressed in the concluding section.

## 2. Modal Parameters of UCBC Structure

The dynamic characteristics of the UCBC structure can be described by using modal parameters in the frequency domain, assuming that the UCBC structure is treated as a linear system. Previous studies have confirmed the validity of this linearization approach through the reliable validation of modal parameters, such as the modal assurance criterion and the coherence function of frequency response functions [12,13,14]. Therefore, the basic model of the UCBC structure can begin from the simplified model presented in Equation (1).

(1) $$M\ddot{X}\left(t\right)+C\dot{X}\left(t\right)+KX\left(t\right)=0,$$

where $X\left(t\right)={\left[\begin{array}{ccc} x_{1}\left(t\right) & \ldots & x_{N}\left(t\right) \end{array}\right]}^{T}$ is the column vector representing the $n^{th}$ degree-of-freedom system; $\dot{X}\left(t\right)$ and $\ddot{X}\left(t\right)$ are the velocity and acceleration of $X\left(t\right)$, respectively; and M, C, and K are defined as the system matrices for mass, damping, and stiffness, respectively. Under a static load condition denoted by $S_{0}$, the damping and stiffness matrices, C and K, are modified to $C_{M}$ and $K_{M}$, respectively, such that the modified linear system of the UCBC structure can be expressed in Equation (2). Here, the mass matrix *M* is assumed to remain constant despite the static load assignment.

(2) $$M\ddot{X}\left(t\right)+C_{M}\dot{X}\left(t\right)+K_{M}X\left(t\right)=S_{0}.$$

If the linear system described in Equation (2) is assumed to be an n-degree-of-freedom system, it can be transformed into a modal model by decoupling all modes into independent modes by using the transformed modal column vector $R\left(t\right)={\left[\begin{array}{ccc} r_{1}\left(t\right) & \ldots & r_{N}\left(t\right) \end{array}\right]}^{T}$ derived from the physical column vector $X\left(t\right)$, as demonstrated in Equation (3). The matrix $U=\left[\begin{array}{ccc} u_{1} & \ldots & u_{N} \end{array}\right]$ comprises all mass-normalized mode shape vectors of the UCBC structure, from $u_{1}$ to $u_{N}$. Consequently, the decoupled linear system in modal coordinates is formulated in Equation (4), where $\dot{R}\left(t\right)$ represents the velocity of $R\left(t\right)$, and $\omega_{n,i}$, $\omega_{n,i}^{M}$, and $\xi_{n,i}$, $\xi_{n,i}^{M}$ are defined as the $n^{th}$ resonance frequency and modal damping ratio, respectively. Moreover, the discrepancies between the original and modified matrices are articulated in Equations (5) and (6), which represent the transformed matrices from the damping and stiffness system matrices, respectively.

(3) $$X\left(t\right)=UR\left(t\right)=\sum_{i=1}^{N}{u_{i}r}_{i}\left(t\right),$$

(4) $$\left[\begin{array}{ccc} 2\Delta \left(\omega_{n,1}\xi_{1}\right) & & zeros\\ & \ddots & \\ zeros & & 2\Delta \left(\omega_{n,N}\xi_{N}\right) \end{array}\right]\dot{R}+\left[\begin{array}{ccc} \Delta {\left(\omega_{n,1}\right)}^{2} & & zeros\\ & \ddots & \\ zeros & & \Delta {\left(\omega_{n,N}\right)}^{2} \end{array}\right]R={M^{-1/2}S}_{0}M^{-1/2},$$

(5) $$\Delta \left(\omega_{n,i}\xi_{i}\right)=\omega_{n,i}^{M}\xi_{i}^{M}-\omega_{n,i}\xi_{i},\;\;$$

(6) $$\Delta \left({\omega_{n,i}}^{2}\right)={\left(\omega_{n,i}^{M}\right)}^{2}-{\left(\omega_{n,i}\right)}^{2}.$$

The term on the right side of Equation (4) is the $n^{th}$ mass-normalized static matrix element (${\hat{s}}_{i}=s_{i}/m_{i}$), and the $i^{th}$ decoupled mode is simplified in Equation (7). As previously derived in the cited study [24], the original SLIF represents the modal column vector, $r_{i}$, with respect to the mass-normalized static load, as illustrated in Equation (8).

(7) $${\hat{s}}_{i}=2\Delta \left(\omega_{n,i}\xi_{i}\right)\dot{r_{i}}\left(t\right)+\Delta \left({\omega_{n,i}}^{2}\right)r_{i}\left(t\right),\;$$

(8) $$I_{f,i}={\left[{\left(2\Delta \left(\omega_{n,i}\xi_{i}\right)\omega_{n,i}\right)}^{2}+\Delta {\left({\omega_{n,i}}^{2}\right)}^{2}\right]}^{\frac{-1}{2}}.\;$$

The $i^{th}$ mass-normalized static matrix element, ${\hat{s}}_{i}$, represents a real value from a physical perspective due to the specific location and nature of the assigned scalar static load, distinct from dynamic excitations such as the harmonic, random, or impulse cases. Given the scalar input condition, the response to the first term, $2\Delta \left(\omega_{n,i}\xi_{i}\right)$, is minimally related to the static load due to the orthogonality between the velocity of $\dot{r_{i}}\left(t\right)$ and the modal response $r_{i}\left(t\right)$. This indicates that the phase of the static load input aligns with the modal response $r_{i}\left(t\right)$. Under static loading conditions, the response of the decoupled system is succinctly formulated in Equation (9).

(9) $$\frac{r_{i}\left(t\right)}{{\hat{s}}_{i}}=\frac{1}{\Delta \left({\omega_{n,i}}^{2}\right)}\;.\;$$

The $i^{th}$ original static load influential factor ($I_{f,i}$, referenced in [24]), was derived from the decoupled expression in Equation (7). However, the $i^{th}$ revised influential factor ($I_{f,i}^{R}$) should be determined from Equation (9), taking into account the phase condition of the static load. Consequently, the revised formulation of the static load influential factor is articulated in Equation (10) and is simplified as a function of variations in the resonance frequency.

(10) $$I_{f,i}^{R}=\frac{1}{\Delta \left({\omega_{n,i}}^{2}\right)}\;.\;$$

Moreover, the physical response $R\left(t\right)$ is also expressed by using the mode summation method as outlined in Equation (11). Here, $d_{i}$ and $\phi_{i}$ represent the $i^{th}$ modal participation factor and the time delay of the UCBC structure, respectively. By integrating Equations (3) and (11), the $i^{th}$ modal column vector ($r_{i}\left(t\right)$) is formulated in Equation (12).

(11) $$X\left(t\right)=\sum_{i=1}^{N}{d_{i}sin(\omega_{n,i}t+\phi_{i})u}_{i},\;$$

(12) $$r_{i}\left(t\right)=d_{i}\sin\left(\omega_{n,i}t+\phi_{i}\right).\;\;$$

In this context, the $i^{th}$ revised SLIF is articulated in Equation (13), and the maximum value of the $i^{th}$ revised SLIF is shown to be proportional to the modal participation factor ($d_{i}$) as presented in Equation (14), assuming a constant mass-normalized static load condition.

(13) $$I_{f,i}^{R}=\frac{d_{i}\sin\left(\omega_{n,i}t+\phi_{i}\right)}{{\hat{s}}_{i}},\;$$

(14) $$I_{f,i}^{R}\propto d_{i}.\;$$

## 3. Identification of Modal Parameters for UCBC Structures

**Figure 1:**
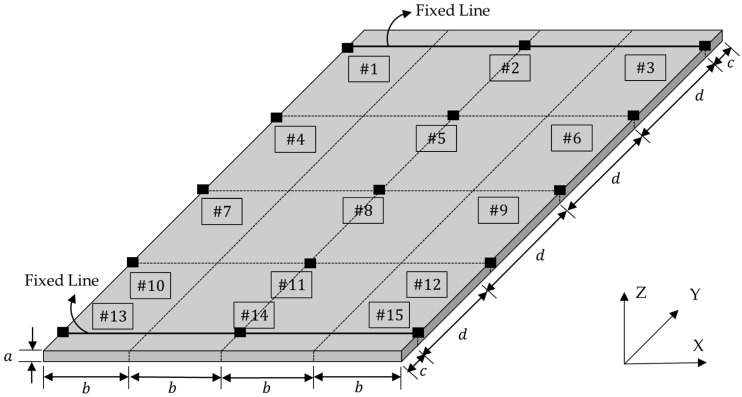
Configuration and boundary conditions of the rectangular specimen: *a* = 3 mm, *b* = 20 mm, *c* = 10 mm, and *d* = 32.5 mm [24].

UCBC specimens were prepared with five different orientations of carbon fiber—0°, 30°, 45°, 60°, and 90°—to investigate the variations in modal parameters in greater detail compared with a previous study [24]. Additionally, an isotropic specimen composed of SUS304 (18Cr-8Ni; POSCO, Pohang, Republic of Korea) was also prepared as a reference. The configuration of the UCBC specimens was a simple rectangle (150 mm × 80 mm × 3 mm), as shown in Figure 1, and the static load was applied in three scenarios, 0 N, 500 N, and 1000 N, under fixed–fixed boundary conditions by using a mechanical fixture. The specimens were fabricated by using the autoclave curing process at a maximum temperature of 125 °C and consisted of 12-layered thin unidirectional pre-impregnated USN 250A (SK Chemical, Seongnam, Republic of Korea). The locations for the accelerometer (model 3225F2; Dytran, Chatsworth, CA, USA) attachments (#1–#15) were chosen in accordance with the methodology of the previous study [24], ensuring consistency in the experimental setup for objective comparisons between the revised and original SLIFs.

The modal testing of all specimens was conducted by using an impact hammer (model 5800B3; Dytran, Chatsworth, CA, USA). Response accelerations were simultaneously recorded to measure the FRFs between them. The modal parameters for all specimens were derived from these FRFs by using Test.Lab software (15A version, Siemens, Munich, Germany), with a designated frequency range of 10 Hz to 6400 Hz. All three static load conditions—0 N, 500 N, and 1000 N—were considered to ensure the reliable extraction of modal parameters by using the PolyMAX algorithm within Test.Lab software. The modal parameters are summarized in Table 1 and Table 2.

**Figure 2:**
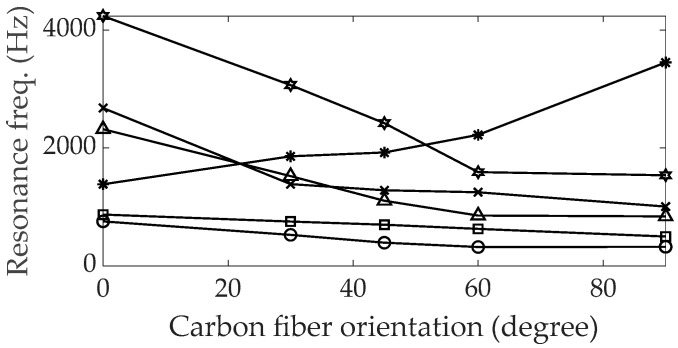
Variations in resonance frequencies by carbon fiber orientation. 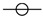: first bending; 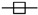: first torsional; 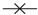: second bending; 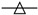: third bending; 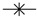: second torsional; 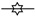: fourth bending.

The variations in the first six identified modes of the UCBC specimens can be observed based on the orientation of the carbon fiber, with consistent increases or decreases in resonance frequency noted in the free–free boundary case of UCBC specimens from a previous study [24]. A similar trend in variations in resonance frequencies was also found for UCBC specimens under fixed–fixed boundary conditions. The first six tracked modes of the UCBC specimens are illustrated in Figure 2.

**Figure 3:**
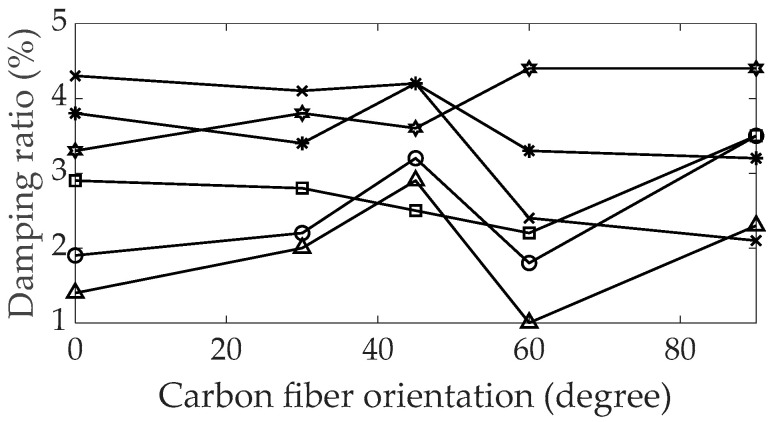
Variations in modal damping ratios by carbon fiber orientation. 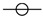: first bending; 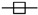: first torsional; 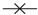: second bending; 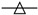: third bending; 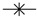: second torsional; 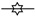: fourth bending.

All five modes (four bending and one torsional) exhibited a decrease in frequency, except for the second torsional mode, which increased as the orientation of the carbon fiber increased. The change in resonance frequency depends on the mode shape of each UCBC specimen and may serve as an indicator to verify the tracked mode shape of each mode. Regarding the modal damping ratio, consistent increases or decreases were not observed, as shown in Figure 3; these findings align with results from previous studies [24]. Compared with the modal damping ratio of the isotropic SUS304 material specimen, which is typically less than 2%, the modal damping ratios of the UCBC specimens ranged between 2% and 4%. These higher values indicate a superior capacity of the UCBC specimens to dissipate vibration energy under dynamic excitation. However, as the orientation of the carbon fiber increased, the damping ratio of UCBC specimens changed in a random manner, making it difficult to predict the variation in modal damping ratio as the orientation of the carbon fiber increased.

The structural stiffness of a UCBC structure is closely dependent on the orientation of the carbon fibers, influencing whether the resonance frequency increases or decreases with variations in carbon fiber orientation. This relationship is illustrated by the transformation of the decoupled stiffness matrix in Equation (3) from the original stiffness matrix in Equation (2). Conversely, the damping characteristics of most UCBC systems are strongly dependent on the combined matrix within the UCBC structure [12,13,14]. The composition of the matrix part in the UCBC specimens remained consistent across different carbon fiber orientations. Consequently, the variations in the orientation of the carbon fibers are not closely linked to the damping characteristics of the UCBC structure, which can explain the observed inconsistency between the orientation of the carbon fibers and the modal damping ratio illustrated in Figure 3. However, accurately identifying the modal damping ratio of the UCBC specimens is essential, given the considerable variations in modal damping ratios across different orientations of carbon fibers.

## 4. Evaluation of Revised SLIF for UCBC Structures

**Figure 4:**
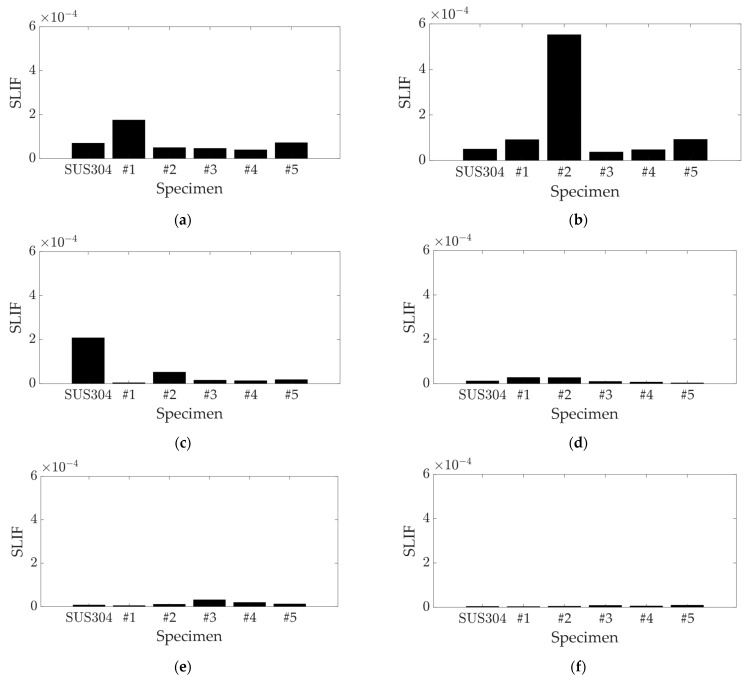
Original SLIF results: (**a**) first mode (first bending); (**b**) second mode (first torsion); (**c**) third mode (second bending); (**d**) fourth mode (third bending); (**e**) fifth mode (second torsion); (**f**) sixth mode (fourth bending).

**Figure 5:**
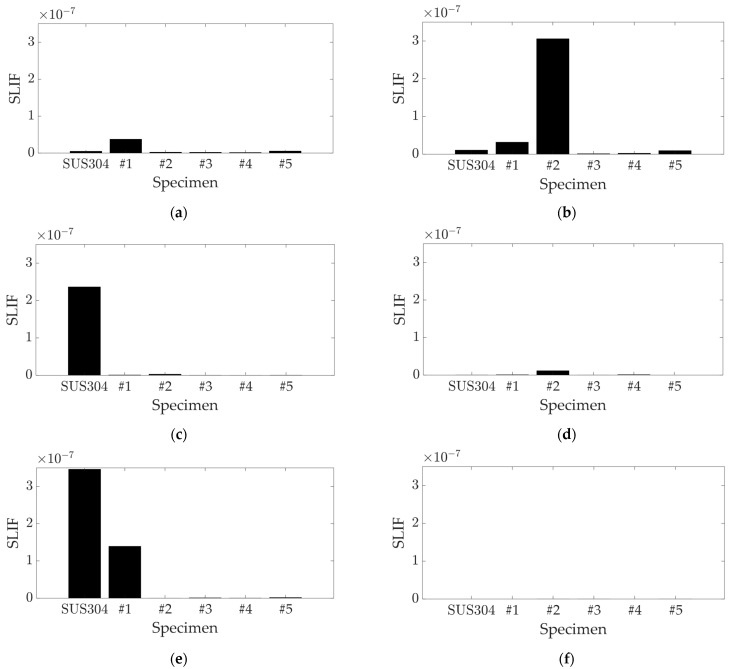
Revised SLIF results: (**a**) first mode (first bending); (**b**) second mode (first torsion); (**c**) third mode (second bending); (**d**) fourth mode (third bending); (**e**) fifth mode (second torsion); (**f**) sixth mode (fourth bending).

The proposed SLIF calculates the sensitivity of the static load on a target structure, considering the real value of the static load case (zero-degree phase), and eliminates the damping-related term in Equation (4), which is orthogonal to the modal response, $r_{i}\left(t\right)$. This term is defined as the discrepancy between the original and the modified damping matrices. The influential factor for each mode was calculated by using both the original and revised formulations, as presented in Equations (7) and (9), respectively. The results for the first six modes are depicted in Figure 4 and Figure 5.

For the first three modes, the UCBC specimens displayed similar original SLIF values (Figure 4) to those found in the previous study [24], but this study considered more detailed cases of carbon fiber orientation. The SLIF for specimen #2 was notably high in the second mode (first torsional) and substantial in the third mode. The results for specimen #2 cannot be directly compared with the previous study, as the 30-degree orientation of UCBC specimens was not previously considered. Specimen #1 showed high SLIF values in the first mode, but these values decreased as the orientation of the carbon fiber increased. The SLIF for the SUS304 specimen was highest in the third mode and decreased in higher modes. Consequently, the most notable high SLIF values were observed for specimen #2 in the second mode, specimen #1 in the first mode, and the SUS304 specimen in the third mode. For the fourth and higher modes, the SLIF values decreased significantly across all specimens, suggesting that the influence of the static load on these higher modes is negligible. Conversely, the revised SLIF for specimen #2 showed high values in the second, fourth, and fifth modes, as shown in Figure 5. The revised SLIF for specimen #1 was the highest in the first mode, and the SUS304 specimen exhibited high revised SLIF values in the third mode. In the sixth mode, the revised SLIF values for all specimens were minimal.

The first three modes showed similar results for both the original and revised SLIF methods, but the influential factor results were inconsistent at higher modes. The high values of the revised SLIF corresponded to very small variations in resonance frequencies: 0.16% for specimen #2 in the second mode, 0.04% for the SUS304 specimen in the third mode, 0.01% for the SUS304 specimen in the fifth mode, and 0.02% for specimen #1 in the fifth mode. The minimal variance in the two specimens (SUS304 and specimen #1) in the fifth mode showed a high value for the revised SLIF but a significantly lower value for the original SLIF. 

The similarity between the two SLIFs can also be analyzed by considering all elements as row vectors at each resonance frequency. A direct comparison between the two SLIF values was not possible due to the different scales of the two influential factors, so the normalization of each SLIF first had to be conducted by dividing it by its 2-norm value. Then, the similarity between the two SLIFs was calculated by using the inner product between the original and normalized SLIFs. If the $i^{th}$ normalized original and revised SLIF vectors are defined as $\vec{S_{i}^{O}}$ and $\vec{S_{i}^{R}}$, respectively, the $i^{th}$ mode similarity between the two SLIFs ($S_{i}$) can be calculated as follows, where the unit of similarity $S_{i}$ percentage (%) and the similarity range from 0 to 100:

(15) $$S_{i}=\vec{S_{i}^{O}}\cdot {\left(\vec{S_{i}^{R}}\right)}^{T}.\;$$

Here, ${\left(\vec{a}\right)}^{T}$ denotes the transpose of $\vec{a}$. The results of the inner product between the two normalized SLIF vectors are summarized in Table 3.

The similarity between the two SLIFs decreased as the resonance frequency increased, which is consistent with previous discussions. The sudden increase in the sixth mode is negligible, due to the small value of the SLIF in that mode. The primary difference arises from the damping term in Equation (4), which was omitted in the revised SLIF method detailed in Equation (9). In the case of the original SLIF, the damping term was multiplied with the resonance frequency, resulting in a rapid decrease in the original SLIF value due to the high value of the resonance frequency, as indicated in Equation (7). Consequently, the original SLIF values became negligible at high frequencies, as illustrated in Figure 4, and these results were far from reflecting the variations in the modal parameters of the specimens of interest. Moreover, the revised SLIF represents a more physically plausible expression because the assigned static load was applied in scalar form (real value). This aspect is a critical point highlighted in discussions about the revised SLIF. The revised SLIF is formulated simply and is inversely proportional to the discrepancy between the squares of the original and modified resonance frequencies, as demonstrated in Equation (5). Therefore, the variations in the resonance frequency of UCBCs under a static load are also inversely proportional to the SLIF. As shown in Equation (8), the magnitude of the decoupled modal response ($r_{i}\left(t\right)$) increases as the $i^{th}$ SLIF value increases. Since the physical response $X\left(t\right)$ is equivalent to the product of the decoupled modal response and the mass-normalized mode shape vector in modal analysis [24], the $i^{th}$ SLIF is ultimately linked to the modal participation factor of the UCBC structure at the $i^{th}$ mode. As a result, the two robust UCBC specimens, #1 and #2, were expected to exhibit distinct responses based on the assignment of uniaxial static load, influenced by the variations in modal participation factors in the first five modes. The influence of static load was also pronounced for the isotropic SUS304 specimen within similar frequency ranges. All these influences of static load are effectively represented by using the proposed SLIF. Further verification of the variations in the modal participation factor of UCBC structures under static load conditions can be achieved through additional experimental testing, which is planned as future work.

## 5. Conclusions

This study developed a revised SLIF that accounts for the in-phase relationship between static loads and decoupled modal responses in UCBC structures. By employing a formulation inversely proportional to the discrepancy between the squares of the original and modified resonance frequencies, the revised SLIF also correlates with the modal participation factor, thereby highlighting that a high SLIF value in any given mode correlates significantly with an increased participation factor in the physical response. Such insights necessitate careful consideration of the pre-assigned static loads in UCBC structures, especially when the SLIF reveals high sensitivity in particular modes. Experimental modal analysis was conducted on UCBC specimens aligned along five distinct fiber orientations, and the modal parameters were meticulously compiled for the first six tracked modes. Both the original and revised SLIFs were calculated from these mode shapes, showing similar values for the initial three modes but revealing that the original SLIF becomes negligible in higher modes due to the amplification of damping terms by resonance frequencies. In the case of the fifth mode, both specimens, SUS304 and UCBC #1, showed minimal variations in resonance frequency (0.02% and 0.01%, respectively), which highlighted the revised SLIF values. However, this observation was not apparent with the original SLIF. The similarity between the two SLIFs was also analyzed by using the inner product of the two normalized SLIF row vectors. The similarity results show values of more than 91.3% for the first three modes, but these values decreased to 70.4% and 22.2% for the fourth and fifth modes, respectively. Therefore, the similarity between the SLIFs demonstrates the superiority of the revised SLIF over the original one.

The testing indicated that two particularly robust UCBC specimens exhibited heightened sensitivity to uniaxial static loads, likely to cause significant variations in modal responses and participation in crucial modes. This sensitivity was also observed in the isotropic SUS304 structure, showing high SLIF values. These findings affirm the effectiveness of the modified SLIF in assessing the sensitivity of assigned static loads on UCBC structures. The refined approach offers a more robust framework for predicting structural responses under static loads, enhancing the predictability and reliability of engineering assessments of composite materials. This study’s novel methodology enhances the utility of the SLIF by aligning it more closely with physical properties and modal interactions, paving the way for more precise engineering applications in composite structural analyses.

## Figures and Tables

**Table 1:** Modal parameters of the SUS304 specimen.

Static Load	0 N		500 N		1000 N	
	Resonance Freq. (Hz)/Mode	Damping Ratio (%)	Resonance Freq. (Hz)/Mode	Damping Ratio (%)	Resonance Freq. (Hz)/Mode	Damping Ratio (%)
SUS304	430.7/B(1)	2.4	443.2/B(1)	1.8	446.8/B(1)	1.9
	932.1/T(1)	1.5	934.4/T(1)	0.7	937.2/T(1)	0.5
	1469.0/B(2)	1.4	1458.8/B(2)	1.1	1468.3/B(2)	1.3
	2148.3/B(3)	1.9	2116.3/B(2)	1.9	2133.3/B(3)	1.4
	2831.9/T(2)	1.9	2841.7/T(2)	1.7	2831.6/T(2)	2.7
	3087.0/B(4)	2.0	3095.9/B(4)	2.1	3125.0/B(4)	2.2

**Table 2:** Modal parameters of the three UCBC specimens.

Static Load	0 N		500 N		1000 N	
	Resonance Freq. (Hz) /Mode	Damping Ratio (%)	Resonance Freq. (Hz) /Mode	Damping Ratio (%)	Resonance Freq. (Hz)/Mode	Damping Ratio (%)
UCBC #1 $\left(\theta_{1}=0{}^{\circ}\right)$	756.4/B(1)	1.9	759.3/B(1)	2.5	759.8/B(1)	2.1
	871.2/T(1)	2.9	875.6/T(1)	3.9	874.4/T(1)	3.5
	1386.7/B(3)	3.8	1397.3/B(3)	4.0	1398.9/B(3)	4.0
	2319.7/B(2)	1.4	2294.2/B(2)	2.9	2312.5/B(2)	3.5
	2679.4/T(2)	4.3	2732.2/T(2)	3.4	2679.9/T(2)	5.7
	3435.7/B	5.9	3386.2/B	4.0	3496.5/B	5.2
	4240.7/B(4)	3.3	4270.0/B(4)	3.4	4267.4/B(4)	3.7
UCBC #2 $\left(\theta_{1}=30{}^{\circ}\right)$	527.5/B(1)	2.2	537.9/B(1)	2.5	546.0/B(1)	2.4
	753.7/T(1)	2.8	753.1/T(1)	2.4	752.5/T(1)	2.8
	1387.1/T(2)	4.1	1361.0/T(2)	4.3	1359.6/T(2)	5.5
	1523.9/B(2)	2.0	1528.8/B(2)	2.2	1529.9/B(2)	2.1
	1858.6/B(3)	3.4	1864.1/B(3)	3.2	1856.1/B(3)	2.9
	2508.0/T	4.0	2499.1/T	5.5	2416.1/T	4.6
	3069.0/B(4)	3.8	3061.8/B(4)	4.2	3080.2/B(4)	4.8
UCBC #3 $\left(\theta_{1}=45{}^{\circ}\right)$	393.6/B(1)	3.2	405.8/B(1)	2.5	419.9/B(1)	2.8
	699.4/T(1)	2.5	707.5/T(1)	1.9	717.7/T(1)	3.0
	1104.9/B(2)	2.9	1120.5/B(2)	2.8	1132.5/B(2)	2.6
	1283.4/T(2)	4.2	1288.3/T(2)	3.9	1295.5/T(2)	4.0
	1923.4/B(3)	4.2	1902.3/B(3)	4.7	1898.2/B(3)	4.4
	2153.2/T	3.3	2155.4/T	3.3	2155.4/T	3.3
	2421.9/B(4)	3.6	2414.7/B(4)	3.5	2405.1/B(4)	2.9
UCBC #4 $\left(\theta_{1}=60{}^{\circ}\right)$	321.1/B(1)	1.8	339.9/B(1)	1.7	357.9/B(1)	1.6
	628.5/T(1)	2.2	638.6/T(1)	2.3	644.0/T(1)	3.0
	853.6/B(2)	1.0	872.7/B(2)	1.8	894.4/B(2)	1.7
	1250.5/T(2)	2.4	1265.0/T(2)	2.5	1270.8/T(2)	2.3
	1591.3/B(4)	4.4	1596.8/B(4)	3.2	1637.6/B(4)	3.2
	1914.0/B	5.0	2031.7/B	3.0	1928.0/B	4.4
	2223.5/B(3)	3.3	2204.8/B(3)	3.0	2230.0/B(3)	4.6
	2366.4/B	4.8	2463.3/B	5.0	2415.6/B	4.9
	3177.4/B	3.3	3172.5/B	4.4	3278.6/B	3.5
UCBC #5 $\left(\theta_{1}=90{}^{\circ}\right)$	324.7/B(1)	3.5	325.8/B(1)	2.2	344.7/B(1)	1.9
	498.2//T(1)	3.5	478.7/T(1)	2.8	487.8/T(1)	2.9
	841.0/B(2)	2.3	901.4/B(2)	2.5	871.0/B(2)	3.3
	1006.7/T(2)	2.1	1012.8/T(2)	3.2	1018.1/T(2)	5.7
	1538.1/B(4)	4.4	1533.0/B(4)	3.4	1560.0/B(4)	2.7
	1653.5/T(3)	6.4	1784.9/T(3)	6.0	1747.5/T(3)	4.8
	2130.7/B	5.5	2293.0/B	3.3	2270.6/B	2.2
	3451.1/B(3)	3.2	3424.4/B(3)	3.1	3412.8/B(3)	3.1

**Table 3:** The similarity of the two normalized SLIFs.

Mode	Similarity (%)
1	91.3
2	98.3
3	96.4
4	70.4
5	22.2
6	87.8

## Data Availability

The original contributions presented in the study are included in the article, further inquiries can be directed to the corresponding author.
